# Comprehensive treatment strategy for improving surgical resection rate of retroperitoneal sarcomas: a histology-specific approach narrative review

**DOI:** 10.3389/fonc.2024.1432900

**Published:** 2024-10-07

**Authors:** Dorian Y. Garcia-Ortega

**Affiliations:** Skin, Soft Tissue and Bone Tumors Department, National Cancer Institute (Mexico), Mexico City, Mexico

**Keywords:** retroperitoneal sarcoma, extended resection, histology-specific approach, reference center, multidisciplinary tumor board (MDTB)

## Abstract

Retroperitoneal sarcoma (RPS) represents a rare and heterogeneous group of malignancies, posing significant challenges in evaluation and management. Surgery, the cornerstone of RPS treatment, critically depends on complete resection for a favorable prognosis. The extent of resection is a crucial determinant of local control and survival. This review delves into the evolution of multidisciplinary management of localized RPS, highlighting the imperative to adapt surgical strategies to tumor histology, location, and patient functional status. We explore the principles of compartmental surgery—an extended first-line approach that involves resecting adjacent viscera for wide negative margins—and its effectiveness across different histological subtypes of RPS and more limited resections for other types. Particular emphasis is placed on the heterogeneity of the disease, as various histological subtypes exhibit distinct biological behaviors. This necessitates a shift away from a one-size-fits-all treatment approach. The review analyzes the role of different surgical strategies, focusing on histological type and location. Additionally, the potential benefits of (neo)adjuvant treatments, such as radiotherapy and chemotherapy, are examined, recognizing their specific histological indications and limitations. This comprehensive review consolidates recent data on surgical strategies and complementary therapies, advocating for a personalized approach tailored to histology. As understanding of the molecular and genetic underpinnings of RPS continues to evolve, so will strategies for its effective management, underscoring the need for global collaboration among specialists in this field to enhance our collective knowledge and treatment methodologies.

## Introduction

Retroperitoneal sarcomas (RPS) are rare malignant neoplasms, constituting less than 1% of all cancers in adults but accounting for approximately 15% of soft tissue sarcomas (STS) (1–4). The incidence of RPS is estimated at 2.7 cases per million people per year, with an equal prevalence among men and women, typically diagnosed between the fifth and sixth decades of life (5). These tumors arise from the retroperitoneal space, an area without defined anatomical boundaries and surrounded by vital structures, which significantly complicates surgical interventions and increases the risk of recurrence even in low-grade tumors (6, 7).RPS are characterized by their histological heterogeneity, with predominant types such as liposarcoma and leiomyosarcoma, representing more than 80% of cases. This variability directly impacts the biological behavior of the tumor and the applicable therapeutic strategies since there are no “low-risk histologies,” even tumors classified as low-grade have high rates of local failure that compromise long-term survival (7, 8). Surgery is the cornerstone of treatment for RPS, being the only curative modality in localized disease. Complete resection (R0/1) has been consistently identified in retrospective historical series as the most important prognostic factor, with recent advances in surgical techniques increasing the R0/1 resection rates to a range of 70-95%. The implementation of compartmental surgery, inspired by principles used for STS of the extremities, has allowed for minimizing incomplete resections (R2) and is currently the recommended approach by leading expert groups. This approach involves en-bloc resection of the tumor and adjacent organs, improving oncological outcomes and reducing recurrence rates; however, it is not a strategy applicable to all sarcomas (8–12).

Despite these advances, the management of RPS continues to face challenges due to the anatomical complexity of the retroperitoneum and the diversity of histological subtypes. This requires a personalized approach based on the specific characteristics of the tumor and the patient. This review discusses these surgical and oncological principles, evaluates existing literature, and outlines strategies to optimize the treatment and survival of patients with RPS.

## Historical perspective

The history of surgical treatment for retroperitoneal sarcomas has evolved remarkably since 1761 when Italian anatomist Giovanni Battista Morgagni first described a lipomatous tumor in his treatise “*De Sedibus et causis morborum per anatomen indagatis.*” Later, in 1829, Lobstein provided a more comprehensive description of these tumors, using the term “sarcoma” for the first time. During the 19th and early 20th centuries, cases were recorded sporadically, often as autopsy findings or during surgical procedures, primarily focusing on pathological descriptions rather than treatment.

Throughout the first half of the 20th century, surgery began to gain recognition as the standard treatment, although techniques and knowledge of the disease were still developing. At the turn of the century, researchers such as J. Dutton Steele and Howard Williams expanded the literature, though with limited cases and infrequent complete resections. It was not until 1933 that, thanks to a series of 46 patients by Judd and Larson at the Mayo Clinic, surgery was established as the primary treatment. However, complete removal was achieved in only a third of these cases (13).

During the 1950s and 1960s, awareness of retroperitoneal sarcomas (RPS) increased, with more extensive case series reported by prominent institutions such as Columbia Presbyterian Hospital and Memorial Hospital in New York. However, the frequency of complete resections remained low, and operative mortality, although reduced, continued to be a challenge. In 1973, a study at Memorial Hospital in New York revealed that only 32.4% of patients with retroperitoneal sarcoma (RPS) underwent complete resection, while 44.1% had partial resection, and 23.5% received only biopsy and radiotherapy. By 1984, another study reported that 38% of RPS patients at the Medical College of Virginia underwent complete resection, with multivisceral resection being necessary in 68% of cases. In 1998, an analysis of 500 patients demonstrated that complete resection significantly reduced the risk of local recurrence compared to incomplete resection, with a postoperative mortality rate of 4%. Finally, in the 2000s, European studies confirmed that extended resection improved recurrence-free survival compared to standard resection, with 5-year recurrence rates of 28% versus 48% (14, 15). Current discussion on resection in retroperitoneal sarcomas focuses on the appropriate extent. Although R0 and R0/1 resections show similar oncologic outcomes, there is evidence suggesting that R0 resection might be superior in some instances; Paik et al (16), in a systematic review, found that R0 margins reduce the recurrence rate (45.5%-52.3% for R0 vs. 66.7%-91.7% for R1). However, the relationship between tumor biology and the extent of resection remains uncertain due to limited data.

In the 1980s and 1990s, advances in surgical techniques and an accumulation of clinical experience led to significant improvements in complete resection rates. The Memorial Sloan Kettering Cancer Center (MSKCC) reported an increase in complete resection rates from 21% to 56% and a notable reduction in operative mortality from 11% to 2%. However, disease recurrence remained high, demonstrating the continued difficulty of managing these tumors even after successful resection (17).

Towards the end of the 20th century, an analysis of 500 patients by Lewis et al. in 1998 at MSKCC underscored the critical importance of complete resection in optimizing outcomes. It highlighted the increasing difficulty of achieving complete resections with each recurrence. This study also emphasized the need to evaluate non-surgical therapies and to develop a more systematic and cooperative approach to studying these rare and complex tumors (6).

Today, the Transatlantic Australasian Retroperitoneal Sarcoma Working Group (TARPSWG), established in 2013, brings together specialists from various disciplines. Starting with eight institutions, the group has expanded its reach to 128 international institutions, fostering global collaboration and translational research in managing retroperitoneal sarcomas. The group has published consensus guidelines and promotes the creation of prospective clinical trials, highlighting a worldwide effort to improve outcomes in this field (18, 19).

## Initial evaluation of primary disease

Patients diagnosed with retroperitoneal sarcomas (RPS) may either exhibit nonspecific symptoms or remain entirely asymptomatic. Tumors are frequently detected incidentally during imaging studies conducted for unrelated reasons. High-quality contrast-enhanced computed tomography (CT) of the abdomen and pelvis is essential for initial evaluation, providing crucial details for surgical planning. In some cases, magnetic resonance imaging (MRI) may offer additional relevant information for delineating the involvement of soft tissues (20–22).

Image-guided core needle biopsy, targeting solid, non-necrotic areas that enhance with contrast using a coaxial technique and an 18-gauge needle to maximize the tissue available for pathological analysis. This biopsy is crucial for ruling out benign pathologies and confirming the histological type of RPS, a necessary step to plan neoadjuvant therapies and other specific management of the tumor histology (22). Although needle biopsy can provide information on the tumor grade, the accuracy of this assessment may be limited, and a detailed pathological evaluation of the resection specimen is recommended. Surgical incisional biopsy is discouraged due to the risk of altering tissue planes for subsequent resection and potential tumor dissemination, as evidenced by a detailed systematic review that examined studies from 1990 to June 2022. This study focused on assessing the incidence of local recurrence and overall survival, comparing patients who underwent preoperative biopsy with those who did not (23). Out of 3192 studies examined, five retrospective cohort studies from the United Kingdom, the Netherlands, and Japan were selected, providing data on biopsy tract seeding. Two of these studies, with a combined size of 572 patients (24, 25), reported no recurrence along the biopsy tract. However, the third study, conducted by Van Houdt et al. (26), which included 498 patients undergoing RPS resection at the Royal Marsden NHS Foundation Trust and The Netherlands Cancer Institute, found a biopsy tract recurrence rate of 2% (5 of 255 patients who underwent preoperative biopsy). These recurrence cases included three grade 2 leiomyosarcomas and two grade 3 liposarcomas. Notably, all recurrences occurred in patients whose biopsies were performed using a transabdominal approach and not a coaxial technique, suggesting a higher risk associated with non-coaxial methods (p = 0.02).

These studies found no significant differences in local recurrence or overall survival between patients who underwent biopsy and those who did not. This finding supports the safety and utility of preoperative biopsy in RPS for appropriate clinical decision-making without negatively impacting long-term outcomes. However, it is crucial to note that the Van Houdt et al. (26) study had a relatively short median follow-up of 38 months, and biopsy tract recurrences occurred between 6 months and seven years after biopsy, indicating the need for prolonged follow-up for a more accurate assessment of long-term risks. These findings suggest that while preoperative biopsy is a safe tool for managing RPS, the technique can significantly influence the risk of complications, particularly biopsy tract seeding, and methods such as the coaxial technique should be considered to minimize this risk.

## Preoperative management of retroperitoneal sarcomas

In the preoperative phase of retroperitoneal sarcoma treatment, it is critical to perform a detailed evaluation in three key areas: the extent of the disease, the patient’s functional performance (including their nutritional status), and the healthcare responsible for the treatment. Staging is crucial for determining the tumor’s extent and planning the surgery effectively. Chest, abdominal, and pelvic computed tomography (CT) is essential for identifying possible visceral metastases, especially in the liver and lungs, which are the most common sites of dissemination for these tumors. Further cross-sectional imaging is also suggested for certain histologic types of retroperitoneal sarcoma that have a propensity to metastasize to the liver, such as leiomyosarcoma. Although positron emission tomography (PET) is not standard for staging these sarcomas, it is being evaluated as a potential tool to provide additional prognostic information about the primary tumor. New technologies such as radiomics and augmented reality are currently under investigation, promising to transform the evaluation of these patients in the future (22, 27–30).

The involvement of a multidisciplinary team with experience and access to adequate facilities is essential for ensuring optimal patient management. Patients with RPS should be assessed and treated by surgical oncologists with specific expertise in sarcomas. These specialists, often part of multidisciplinary teams that include medical oncologists, radiation oncologists, pathologists, and radiologists, significantly enhance patient outcomes. It has been suggested that a minimum volume of 10 to 13 RPS cases annually is necessary to maintain competency in managing these complex and rare tumors (22, 27, 28).

RPS surgery, typically performed after confirming the absence of metastatic disease, presents unique challenges due to the often large size of these tumors and their proximity to critical organs and structures. Therefore, preoperative planning is an integral component of the surgical process, with preparations ranging from consultations with other surgical specialists to coordination with anesthesiology to anticipate intraoperative needs such as transfusions and venous access (8, 12, 20, 22, 24, 31, 32).

Particular attention must be given to the patient’s comorbidities. Comprehensive medical evaluations are necessary to determine the viability of extensive procedures, such as ipsilateral nephrectomy in patients with compromised renal function or interventions to enhance cardiopulmonary function in those with significant preexisting conditions. Moreover, careful nutritional evaluation and optimizing the patient’s protein-caloric status are essential to improve postsurgical outcomes. This meticulous and personalized preparation not only facilitates the execution of the surgery but also maximizes the chances of a successful outcome, minimizing complications and enhancing the patient’s quality of life after the intervention (33–35).

## Surgical approach

In the surgical management of retroperitoneal sarcomas, selecting the appropriate surgical technique is essential to ensure optimal access to both the neoplasm and adjacent critical anatomical structures. Generally, an extensive midline laparotomy is preferred because it effectively exposes the tumor and critical vascular structures, including the aorta and inferior vena cava (IVC). However, depending on the tumor’s location and size, adjustments to the surgical approach may be necessary. This could include considering lateral or thoracoabdominal incisions to accommodate the surgeon’s preferences and the unique aspects of the case (8, 13, 20, 22).

The main objective of surgery for retroperitoneal sarcomas is the en-bloc resection of the tumor along with affected organs, aiming for a complete resection. Resections are classified as R0, with total excision and margins free of disease at a microscopic level; R1, where the margins are microscopically positive; and R2, where the resection is incomplete. Studies indicate that a partial or R2 resection, which often leads to tumor rupture or leaves visible tumor residue, significantly worsens oncological outcomes and increases mortality compared to complete resections; achieving R0 margins is ideal, though it presents a significant challenge in practice, especially considering the size of the tumors and the anatomical complexity of the retroperitoneal space. The complete evaluation of margins in large tumors is challenging, and while R0 resections are associated with better oncological outcomes, this advantage may be influenced by the presence of smaller tumors (7, 22, 27, 36).

In an aim of R0 resection, preoperative planning should include anticipation of tumor involvement in organs and structures, which may require simultaneous resections. Recent studies from sarcoma centers show that in 58-87% of primary PRS cases, surgeons perform resections of one or more organs, commonly including ipsilateral nephrectomy and partial colectomy. Resections of major vascular structures, such as the IVC, although less common, are feasible with appropriate planning and support (7, 37, 38).

For tumors on the left side, surgeons may need to perform a distal pancreatectomy and splenectomy. Conversely, tumors on the right may require a pancreaticoduodenectomy, though this procedure is rare. Tumor laterality and specific characteristics dictate these surgical decisions, underscoring the need for a personalized and meticulously planned approach in RPS surgery. For right upper retroperitoneal tumors that displace the liver, we recommend a thoracoabdominal incision. This technique allows exhaustive control from the inferior vena cava to the right atrium. During surgery, access can be improved by placing a rolled towel under the tumor side of the patient or by positioning the patient partially on their side, with the arm on the same side elevated on support (39, 40).

The first surgical step involves the release of the root of the mesentery, followed by separation of the omentum from the colon and division of the transverse colon, which facilitates access to the major vessels. It is essential to initiate tumor release from the center outward to adequately prepare vascular structures and minimize tension, thereby reducing the risk of vascular tears. It is most effective to begin vascular dissection from the iliac vessels to the proximity of the aorta or vena cava using a subadventitial technique. The adventitial layer is preserved on the tumor side as an anatomical barrier. The primary vascular branches are ligated near the edge of the tumor, including the gonadal and renal vessels. If the renal artery cannot be divided before the renal vein due to the size of the tumor, clamping can be used to stop the flow temporarily. At the same time, access is improved, dividing and then ligating the renal vein. An endoscopic stapling device with vascular clips may be helpful when exposure is limited.

Regarding nerve management, the femoral nerve is located just above the inguinal ligament, accessing through the fascia of the psoas muscle. If the psoas is compromised, it is resected while preserving the femoral nerve. Other sensory nerves, such as the genitofemoral, ilioinguinal, iliohypogastric, and lateral femoral cutaneous nerves, are preserved to the extent possible to reduce the risk of postoperative dysesthesias. Finally, the tumor is removed en bloc along with adjacent structures such as the kidney, the colon on the same side and its mesocolon, and the psoas aponeurosis or the entire muscle. The inner part of the lateral abdominal wall and the peritoneum of the diaphragm are also preserved on the side of the tumor and are resected if infiltrated. After removing the tumor, the diaphragm is reconstructed, and the greater omentum can be used to fill the surgical bed, avoiding displacement of the abdominal viscera (39, 41).

## Compartmental resection

The surgical approach should be tailored to each case’s specific, considering factors such as tumor boundaries, recurrence patterns, and the risk of systemic failure. Compartmental resection is the standardized surgical technique for managing retroperitoneal sarcomas; this is particularly applicable in well-differentiated liposarcoma (WDLPS), dedifferentiated grade 2 (DDLPS G2), and grade 3 (DDLPS G3surgical methods may vary significantly for other histotypes, such as leiomyosarcoma, solitary fibrous tumor, or malignant peripheral nerve sheath tumor (MPNST).

The compartmental resection technique involves the removal of the visible tumor and potentially compromised nearby organs, structures, and surfaces, such as the psoas fascia, to ensure circumferential soft tissue-free margins (8, 20, 22, 39–41). While this method has faced controversy and has not been universally adopted, studies have shown its effectiveness (42). For instance, Gronchi et al. reported that compartmentectomy reduces recurrences threefold at three years (10% vs. 50%) (43). Similarly, Bonvalot et al. noted a reduction in local recurrence from 48% to 28% at five years (44). Recent studies also indicated that compartmentectomy can decrease recurrence rates from 42.3% to 20% (p = 0.007) (12).

This procedure is particularly suitable for treating liposarcoma, thoroughly considering the patient’s clinical status, including comorbidities and expected oncological outcomes, before determining the extent of resection required. If the organs do not show clear evidence of tumor involvement, the decision to resect them depends on whether they can be preserved without significant risks of complications. For example, the rate of Acute Kidney Injury (AKI) post-nephrectomy is 14.8% versus 4.3% without nephrectomy, with a notable reduction in the first postoperative days and only 0.3% of patients requiring permanent dialysis (45). The complication rate of post-colectomy intestinal anastomotic/fistula is 6% (46).

Recent studies on the frequency of microscopic infiltration in resected organs have informed surgical decisions, finding that histological invasion is frequent and varies by organ and histological type (absent (HOI-0), perivisceral (HOI-1), initial (HOI-2), advanced (HOI-3)). It is also associated with a higher risk of recurrence and death, as demonstrated by the study by Improta et al., where patients with HOI-3 had significantly shorter overall survival (HOI-3 vs HOI-0/HOI-1 HR 2.92; p = 0.012) and disease-free survival (HOI-3 vs HOI-0/HOI-1 HR 2.23; p = 0.045) (47). Surgical decisions must balance potential morbidity against essential oncologic principles, such as maintaining tumor integrity to ensure complete en-bloc resection.

Adopting a histology-based surgical approach has gained recognition for soft tissue sarcoma surgery in primary disease, particularly when anticipating the tumor’s origin and local extent based on its histological type. For instance, a leiomyosarcoma originating in the inferior vena cava (IVC) will require a surgical strategy focused on this structure. Regarding liposarcomas, the necessity of performing an extended resection of adjacent “at risk” fat continues to be debated, especially distinguishing between well-differentiated liposarcomas, which are less invasive, and those with dedifferentiated components, which are more aggressive. The surgical strategy for treating retroperitoneal sarcoma (RPS) must be tailored individually, considering the histology of the tumor and its specific risk of recurrence, which varies significantly among different types. For instance, leiomyosarcoma often does not require extensive resection due to its tendency to metastasize distally rather than recur locally.

In contrast, tumors such as solitary fibrous tumors, which have a low risk of recurrence, may be managed with less invasive surgical approaches. The surgical approach for RPS focuses on removing the visible tumor and includes a detailed assessment of the risk for multifocal disease and potential future recurrences. This comprehensive evaluation helps define the necessary extent of resection based on each tumor’s unique characteristics and behavior (20, 22, 27, 40).

A study published in EJSO by Willis (48) demonstrates that patients with retroperitoneal sarcoma (RPS) report a relatively high quality of life, even after undergoing multiple and multivisceral resections. The study by Zhuang et al. showed that while aggressive surgical approaches may impair quality of life within the first six months post-operation, long-term quality of life is similar to that of patients who underwent simple tumor resection. Additionally, the study found that as the postoperative interval increased, all indicators improved in patients who underwent multivisceral resection, whereas no significant improvement was observed in patients without MVR (49).

## Sarcomas from the right side

Surgical treatment of retroperitoneal sarcoma located on the right side requires special considerations, particularly regarding the possible involvement of the inferior vena cava (IVC), pancreas, and duodenum. The main goal of this intervention is to achieve adequate exposure to the tumor. This is accomplished by performing meticulous dissection through the connective tissue and carefully separating the tumor from these vital organs. Extensive resections are avoided unless there is clear macroscopic involvement. Additionally, it may be necessary to mobilize the right liver by dividing the coronary and falciform ligaments and rotating the liver to the left; subsequent steps include performing a coloepiploic separation and dividing the transverse colon to the right of the middle colic artery and the distal ileum. The right colic vessels are isolated proximally from the superior mesenteric vessels, and the mesocolon is separated from the main vessels. A Kocher maneuver is performed to free the duodenum and head of the pancreas, facilitating complete IVC access. This process is especially critical for right-sided tumors, as the duodenum and head of the pancreas often adhere closely to the tumor surface, sometimes leaving only a thin layer over the tumor, if any margin at all (39, 41).

Preservation of the duodenum and pancreas is prioritized and occurs in only 1.4% of cases. Given that pancreaticoduodenectomy has not shown significant improvements in disease-free survival and is associated with a high rate of complications—including a third of cases developing pancreatic leaks and up to a mortality rate of 3.4%—it is generally avoided. However, if duodenal perforation occurs during dissection at the pancreaticoduodenal junction due to wall thinning—a result of compression or tumor invasion—partial resection may be considered. In exceptional cases of severe infiltration, a pancreaticoduodenectomy could be justified, and in these cases, imaging can demonstrate up to 80-85% invasion microscopically (50). In cases where resections require vascular involvement, morbidity increases significantly (54% vs 25%; p < 0.0001). This is also associated with longer surgical times (480 minutes vs 330 minutes; p = 0.001), a higher risk of relapse (local: 45% vs 24%, p = 0.05; distant: 20% vs 0%, p = 0.04), and an increased risk of death (60% vs 81%; p = 0.05) (51).

## Sarcomas from the left side

In the management of retroperitoneal sarcomas located on the left side, the process begins with coloepiploic separation. Subsequently, the transverse colon is divided on the left side of the middle colic artery. The inferior mesenteric vein is ligated along the lower edge of the pancreas. When the tumor is confined to the left superior retroperitoneum, the left superior colic artery is ligated, and the descending colon is divided at its junction with the sigmoid colon. This technique possibly preserves the inferior mesenteric artery. The inferior mesenteric artery is ligated for tumors primarily located in the lower part of the left retroperitoneum, and the sigmoid colon is divided at the rectosigmoid junction. The left mesocolon is then separated from the main vessels and preserved as an anterior resection margin. The duodenojejunal junction may be displaced but not constantly invaded; it may detach from the tumor surface, which usually remains covered by the root or medial edge of the left mesocolon. If the tumor has invaded or is tightly adhered, the third and fourth portions of the duodenum and the proximal jejunum just distal to the ligament of Treitz are divided. This leaves the duodenojejunal junction attached to the surface of the tumor. During reconstruction, a side-to-side anastomosis is performed between the second portion of the duodenum and the remaining proximal jejunum. For tumors confined to the left lower retroperitoneum (i.e., below the transverse mesocolon), the distal pancreas and spleen are separated from the top of the tumor. The tumor remains covered by the transverse mesocolon and the lateral wall and is rotated medially to achieve good exposure. For tumors extending into the left upper retroperitoneum, the distal pancreas is divided. The splenic artery and vein are ligated, and the aorta is dissected up to the diaphragmatic hiatus. The spleen is mobilized en bloc with the upper portion of the tumor. A segment of the posterior aspect of the diaphragm may be resected to facilitate safer tumor mobilization. In such cases, the distal pancreas and spleen form part of the anterior margin of the specimen, and up to 42.4% demonstrate microscopic invasion in the absence of frank macroscopic invasion. It is important to note that grade B pancreatic fistulas have been documented in 18.2% of cases. These structures, including the diaphragm, can serve as the upper margin of the specimen (39–41, 52).

## Management of pelvic retroperitoneal sarcomas

Management of pelvic retroperitoneal sarcomas, which constitute approximately 18% of all retroperitoneal sarcomas, requires meticulous evaluation of tumor characteristics and the complex network of anatomical structures of the pelvis. Comprehensive staging, using abdominopelvic computed tomography (CT) or pelvic MRI, is crucial, especially for histological subtypes such as well-differentiated (WD-LPS), dedifferentiated (DD-LPS) liposarcoma, leiomyosarcoma (LMS), and solitary fibrous tumors. This detailed staging is essential for personalized surgery to preserve the anatomy and function of the pelvic organs while achieving optimal tumor resection (53, 54).

The extraperitoneal pelvic cavity, bounded by the parietal peritoneum, pelvic floor, pubis, inguinal ligaments, and sacrum, presents unique challenges in obtaining wide surgical margins due to the proximity of vascular, bony, and visceral structures. Pelvic sarcomas often exert pressure on organs such as the bladder, prostate, seminal vesicles, or ureters. Although joint resection of these organs with the tumor is rarely justified due to the infrequency of direct invasion, partial resection of the bladder may be necessary to preserve its functionality when bladder invasion occurs. Involvement of the mesorectum may require including the rectum in the resection, and in severe cases, abdominoperineal resection may be necessary. Pelvic recurrences, often more complicated, tend to require more extensive visceral resections (36, 54).

Intraoperative identification of ureters is complex, and preoperative ureteral catheters or nephrostomies are frequently required to manage obstructive hydronephrosis. This is followed by resection of the distal part of the ureter and bladder reimplantation. Tumor fragmentation increases the risk of local recurrence and reduces survival; therefore, large, recurrent, or tumors adherent to the bony pelvis may benefit from neoadjuvant or intraoperative chemotherapy and radiotherapy. This integrated and meticulous approach is crucial for managing pelvic retroperitoneal sarcomas, combining advanced surgical techniques with adjuvant treatment strategies to maximize survival and preserve the patient’s quality of life. In the TARPSWG RPS report, the series demonstrated a local recurrence-free period of 74.1%, a distant recurrence-free period of 79%, and an overall survival (OS) of 67% (55). The upcoming PELVISARC results show an OS of 69.6%, a local recurrence-free period of 62.7%, and a distant recurrence-free period of 66.5%, with leiomyosarcoma being the most reported histology, prompting considerations for a differentiated approach to maximize outcomes.

For RPS with pelvic extension, adhesion to structures such as the rectum and bladder peritoneum and possible extension into the sciatic or obturator notches demands a detailed preoperative evaluation for optimal surgical planning. Preservation of pelvic organs and nervous structures is crucial unless they are directly invaded by the tumor. Sarcomas arising from the psoas muscle and parietal sarcomas present unique challenges due to their deep location within muscle tissue, where the fascia acts as a natural protective barrier. In some instances, an extraperitoneal approach may be appropriate. However, adopting a transperitoneal approach that ensures meticulous vascular control is crucial for more aggressive histologies, such as undifferentiated pleomorphic sarcoma (UPS) (36, 53, 54, 56).

## Why adopt a position based on histology?

The adaptation of surgical strategies based on histology is critical in the treatment of retroperitoneal sarcomas (RPS) because overall survival (OS) heavily depends on local control, especially for low- and intermediate-grade tumors where extended surgery offers significant benefits. However, the approach becomes more complex with high-grade tumors, which have a higher propensity for distant metastases (DM). In these cases, the primary goal is to achieve a complete resection (R0), often supplemented with systemic treatments to minimize DM risks (16, 51). This strategy has been detailed in studies by A. Gronchi and others in 2016 (55) and Callegaro et al. in 2021 (57). These studies differentiate the recurrence patterns between well-differentiated liposarcoma (WDLPS) and grade 2 dedifferentiated (DDLPS G2) versus grade 3 dedifferentiated (DDLPS G3) and leiomyosarcoma (LMS), underscoring that DM critically impacts OS. In LMS, the delineation of tumor borders and the relationship with neighboring organs are more defined, making the quality of the initial surgery crucial for safely preserving non-infiltrated structures and maintaining the radical nature of the procedure. Thus, implementing first-line extended surgery in treating RPS should consider the histological subtype and the tumor’s expected biological behavior and recurrence patterns. Moreover, assessing the tumor in the context of the patient’s overall health is essential, as it influences the feasibility of undergoing extensive surgeries and adjuvant treatments (36, 40, 56, 58, 59).

**Figure 1:**
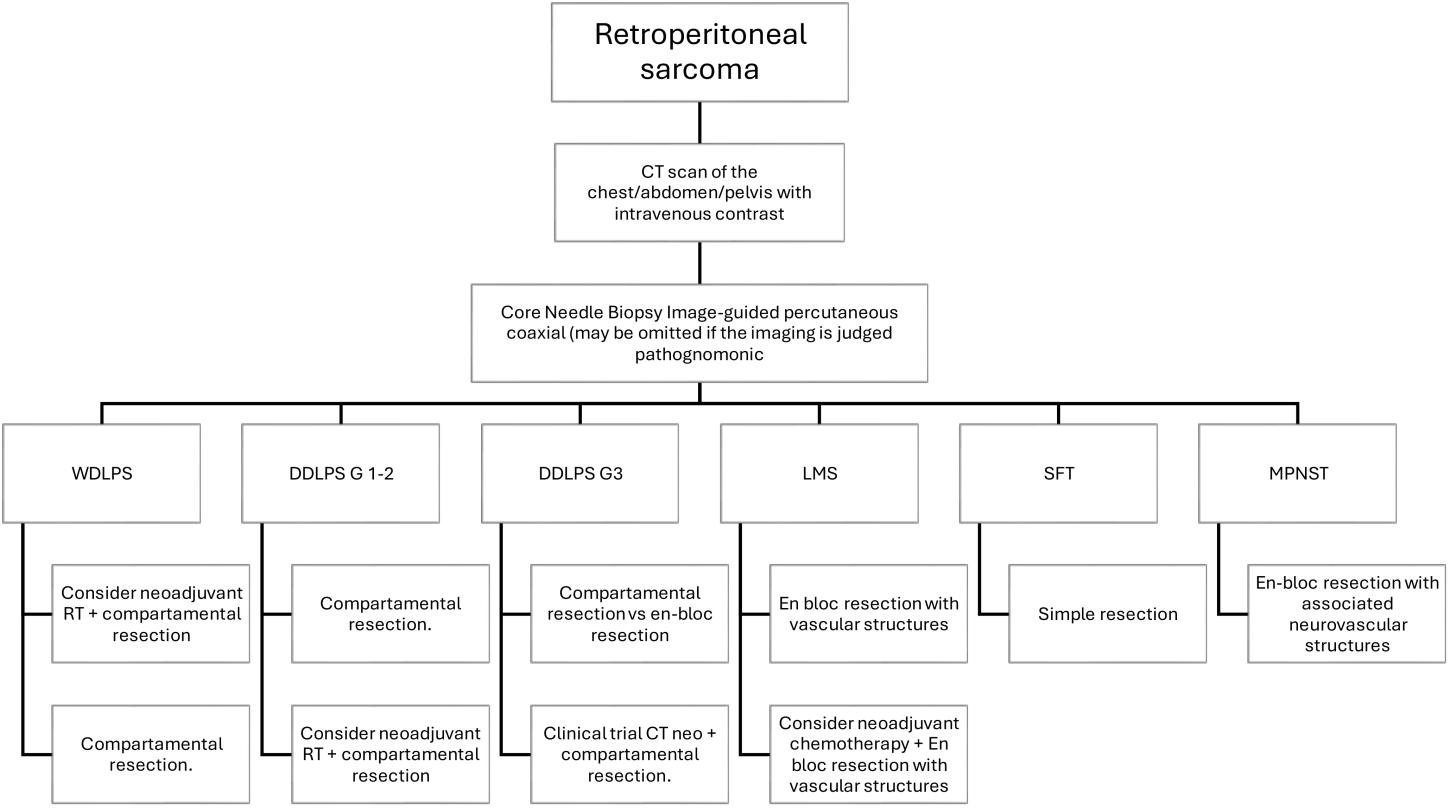
Proposed algorithm for evaluation and management of localized retroperitoneal sarcoma according to histology.

The treatment of retroperitoneal sarcomas (RPS) is significantly enhanced by a multidisciplinary team (MDT) approach, mainly when patients are treated within specialized sarcoma centers (NSC) (60). A study involving 2,945 patients revealed that those who underwent their first surgery at an NSC had notably better outcomes than those treated at out-of-network centers. Specifically, 41.9% of patients in NSCs achieved first R0 resections, a stark contrast to the 12.3% in out-of-network centers. Additionally, the overall survival (OS) was significantly superior for patients treated within NSCs, with a 2-year OS of 87% compared to 70% for those treated elsewhere (p < 0.001). Multivariate analysis confirmed that surgery within an NSC independently predicted better OS, showing a twofold reduction in the odds of death (61). Beyond clinical benefits, the multidisciplinary approach also optimizes healthcare resources, reducing treatment costs by approximately 10-15% due to better therapeutic planning and avoiding unnecessary procedures. These findings underscore the critical importance of MDTs in improving oncological outcomes and enhancing the efficiency of healthcare for RPS patients (22, 27, 28, 60) **Table 1**; **Figure 1**.

**Table 1:** The histological type of retroperitoneal sarcoma, with its associated dissemination pattern, 5-year disease failure rate, and surgical implications.

RPS Histology	Proportion	Relapse pattern	Surgical Management	5-Year OS	5-Year LR	5-Year DR
**WDLPS**	23%-28%	Local	Extended en bloc resection requires resection of ipsilateral retroperitoneal fat	87%	19%-39%	0%
**DDLPS** **G2** G3	32%-43%	Mixed	Extended en bloc resection requires resection of ipsilateral retroperitoneal fat	54% 41%	44% 33%	10% 44%
**LMS**	18-23%	Distant metastasis from early stages of the disease	En bloc resection with vascular structures may preserve adjacent critical structures	58%	6%-16%	56%
SFT *Risk class* Low Moderate High	5%-6%	Mixed None Mixed Mixed	Simple resection	85%	5%-8%	17%
**MPNST**	3%-23%	Mixed	En-bloc resection with associated neurovascular structures may preserve adjacent critical structures	67%	20%-35%	13%

OS, overall survival; LR, locoregional recurrence; DR, distant recurrence.

## Liposarcoma

Retroperitoneal liposarcomas are the most common type of sarcoma in this location. Among these, well-differentiated liposarcoma (WDLPS) represents 50–60% of cases, followed by dedifferentiated liposarcoma (DDLPS) at 30-37%, and other liposarcoma subtypes comprising the remaining 7% (62–64).

WDLPS is a low-grade tumor primarily composed of proliferating mature adipocytes. Despite its relatively indolent behavior, WDLPS has a high propensity for local recurrence, with rates reaching up to 43% at 8 years. Although it rarely metastasizes, approximately 20% of cases may dedifferentiate into a higher-grade liposarcoma. The overall survival (OS) at 5 years for WDLPS is 87%. DDLPS is a high-grade tumor that can arise *de novo* or as a progression from a WDLPS. This subtype is significantly more aggressive and has a high metastatic potential. DDLPS can exhibit heterologous components, such as osseous, muscular, or neurogenic differentiation, although these components do not significantly impact the prognosis. The local recurrence rate for DDLPS is 40%, and the rate of distant metastasis ranges from 15-20%. The overall 5-year survival rate for DDLPS varies between 44% and 53%, with worse outcomes in retroperitoneal cases. Specifically, the 5-year OS is 54% for grade 2 dedifferentiated liposarcoma (G2 DDLPS) and 41% for grade 3 dedifferentiated liposarcoma (G3 DDLPS) (55).

Myxoid liposarcoma (MLS) is rare in the retroperitoneum. Some authors suggest that due to its rarity in this location, these tumors should be considered metastatic until primary lesions are ruled out, particularly in the extremities. When MLS presents in the retroperitoneum, its prognosis is variable, depending on the presence of a round cell component, which increases the metastasis rate to between 30-40% (65).

Pleomorphic liposarcoma (PLS) is the rarest and most aggressive liposarcoma subtype, with the retroperitoneum being an unusual site of occurrence. At diagnosis, PLS may present with metastases in 30-50% of cases, most commonly to the lungs (66).

The diagnosis of retroperitoneal liposarcomas relies on a combination of imaging studies and histopathological evaluation. The treatment of these tumors requires a highly specialized multidisciplinary approach. Surgery remains the cornerstone of management to achieve complete resection. However, the anatomical complexity of the retroperitoneum often makes it challenging to obtain negative margins, highlighting the importance of surgical expertise and intraoperative decision-making informed by tumor biology. Preoperative radiotherapy is the preferred modality for reducing local recurrence, although the proximity of vital organs limits its use. Chemotherapy, while not a standard treatment, may be considered in specific cases, particularly in more aggressive subtypes or in tumors that are not fully resectable. Oncological outcomes in retroperitoneal liposarcomas are closely related to the histological subtype, tumor grade, and the effectiveness of local control. Although WDLPS has a relatively high survival rate, the dedifferentiated and pleomorphic subtypes present a significantly worse prognosis, with high morbidity associated with local recurrence and metastatic progression. Local recurrence remains the most significant clinical challenge and the leading cause of death in patients with retroperitoneal liposarcomas (22, 27, 28).

As discussed in previous sections, the management of these cases requires a personalized therapeutic strategy based on the tumor’s clinical and molecular characteristics, with the goal of optimizing long-term outcomes for patients.

## Leiomyosarcoma

Retroperitoneal leiomyosarcoma is a rare mesenchymal tumor with an inferior prognosis, occurring predominantly in women with a ratio of 3:1 (67). These tumors are more common in the fifth or sixth decade of life, but they can affect any age group. IVC leiomyosarcomas, which arise predominantly in this vein and constitute 50% of all venous leiomyosarcomas, are classified according to the affected segment of the IVC: inferior, middle, and superior, with the middle segment being the most frequently involved. The renal vein is a critical anatomical landmark for this classification (68).

These are slow-growing tumors that exhibits three patterns of growth: extraluminal (62%), intraluminal (5%), and a combination of both (33%). Multiplanar computed tomography (CT) with sagittal and coronal reconstructions is essential to reveal the craniocaudal extent of the tumor. Typical CT features include an irregularly distended IVC containing a lobulated soft tissue mass with heterogeneous enhancement, reflecting the internal hemorrhage and necrosis these tumors often exhibit (69).

Differentiating IVC leiomyosarcomas with an extraluminal growth pattern from other retroperitoneal venous leiomyosarcomas can be diagnostically challenging. Preoperative imaging should detail the tumor’s size, location, relationship to surrounding structures, degree of IVC involvement, relationship to the renal and retrohepatic veins, and any intraluminal tumor components. Defining the lumbar vessels and collateral veins in the retroperitoneum is crucial to anticipate possible blood loss during surgery (69, 70).

Due to the rarity of these tumors, data to guide optimal oncologic treatment are limited; however, a multidisciplinary approach is generally recommended. Surgical resection of the primary tumor is considered the only potentially curative treatment, with 5- and 10-year survival rates of 49.4% and 29.5%, respectively. The surgical strategy depends on the location and extent of the tumor. Several important surgical considerations must be considered for primary leiomyosarcomas of the inferior vena cava (IVC) located below the renal vessels. In many patients, collateral venous pathways develop, which should ideally be identified preoperatively using imaging modalities such as CT or MRI venography. Ligation of the IVC is typically performed after tumor excision. However, if there is significant preoperative venous flow, reconstruction may be required using a prosthetic graft, such as polytetrafluoroethylene (PTFE). The graft size varies depending on the native IVC, usually between 16 and 22 mm. In cases where the surgical site is potentially contaminated, such as when bowel resection is necessary, cadaveric tissue and autologous tissue grafts offer distinct advantages. These materials may reduce the risk of infection, particularly in a contaminated field, as reported in multiple surgical series. For leiomyosarcomas located above the renal vessels and involving the renal vein ostia, surgical exposure can be achieved by mobilizing the right lobe of the liver or via an anterior approach by cutting the caudate lobe. The anesthesia team should be alerted before surgery, and clamping of the IVC above the tumor is necessary to assess the patient’s hemodynamic response. If the patient tolerates this maneuver, segmental resection of the IVC can be performed without reconstruction. In cases where the right kidney is resected, the left renal vein should be divided proximally to the left gonadal vein to preserve venous drainage. Studies have shown no significant differences in complication rates, postoperative morbidity, or 5-year overall survival between patients undergoing IVC reconstruction versus those with ligation. However, patients requiring IVC reconstruction were more likely to need ICU admission (83% vs. 33%; p = 0.0257). Those with IVC ligation tended to develop postoperative lymphedema (35% vs. 0%; p = 0.1615), which resolved in most cases (71, 72).

Kidney autotransplantation can also be utilized to preserve renal function, especially in situations where the renal hilum is involved, as highlighted in studies demonstrating the feasibility and success of this approach. For leiomyosarcomas extending above the hepatic veins, hepatic resection may be necessary if the tumor involves the retrohepatic IVC. This procedure requires total hepatic vascular exclusion and venovenous bypass, which are complex but can be lifesaving. The retrohepatic IVC can then be exposed using a liver suspension technique, particularly useful in avoiding liver congestion. Tumors in the upper segment may necessitate extracorporeal circulation to ensure complete resection and reduce intraoperative blood loss. Studies indicate that despite the complexity of these procedures, with proper patient selection and surgical expertise, long-term outcomes can be favorable (70, 71, 73).

Postoperative complications occur in 18% to 30% of patients and commonly include lower extremity edema and renal failure. Leiomyosarcomas of the lower segment of the IVC is associated with a lower incidence of postoperative complications than those of the upper segment. Additionally, in patients with advanced disease, surgical resection of metastatic disease, such as pulmonary metastasectomy or liver resection, may improve overall survival (68, 71, 73). Although surgery is technically demanding, it remains the primary treatment approach for all patients with localized disease. Recognizing the significant risk of distant metastasis is critical, especially in larger or higher-grade tumors. A recent study also highlights that, in patients with tumors originating in the inferior vena cava, the degree of macroscopic vascular invasion is a crucial clinical predictor of MD (74).

The role of neoadjuvant chemotherapy and radiotherapy in reducing tumor size and increasing resectability remains uncertain, although their use is advised. Chemotherapeutic agents used include dacarbazine, doxorubicin, cyclophosphamide, ifosfamide, and cisplatin, although there is no standard regimen, and management varies depending on patient characteristics. The 5-year disease-free survival for patients with leiomyosarcoma of the IVC is 6%, while the overall survival rate reaches 55% (68).

## Solitary fibrous tumors

Solitary fibrous tumors (SFTs) were described morphologically in 1931 by Klemperer and Rabin as pleural neoplasms (75). Initially termed “solitary (localized) mesothelioma of pleura” by Stout and Murray in 1942, these tumors were later renamed “solitary fibrous tumor” in 1951 by Stout and Hamidi. The term “Hemangiopericytoma” (HPC) was introduced by Stout and Murray in the same year while describing a similar tumor series. These terms reflected the evolving understanding of these tumors, which were considered part of a histomorphological spectrum until their unifying molecular signature was discovered. The defining molecular feature, identified as the recurrent fusion of NAB2 and STAT6 genes on chromosomal region 12q13, solidified the classification of SFT and HPC as ends of a spectrum of a single tumor entity (76). This was officially recognized in the 4th edition of the World Health Organization (WHO) Classification of Tumors of Soft Tissue and Bone, which consolidated these tumors into a single entity. The WHO classifies SFT as a fibroblastic neoplasm with intermediate (rarely metastasizing) behavior. Interestingly, the WHO Classification of Tumors of the Central Nervous System describes extra meningeal SFT and HPC as a single group. Still, different histologic grades are retained along with their distinct names (77).

SFTs can occur across a wide range of ages but are more common in the fifth and sixth decades of life and are rare in children and adolescents. The mean age of presentation for extrapleural tumors is 50.3 years. There are no known specific risk factors for the development of SFTs. SFTs are generally slow-growing, asymptomatic tumors, often discovered incidentally in imaging studies. They can vary in size from 1 to 40 cm, with an average length of 5 to 8 cm. Some may present with Doege-Potter syndrome, a paraneoplastic hypoglycemic syndrome due to the excessive production of insulin-like growth factor II (IGF2) (18, 76).

According to Demicco et al., predictors of time to metastasis and tumor-related death include patient age, tumor size, mitosis rate, and necrosis. The Demicco model classifies risk as follows: patient age (score 0 for <55 years; 1 for ≥55 years), tumor size (score 0 to 3, from <5 cm to ≥15 cm), mitotic frequency of tumor cells (score 0 to 2, from 0/mm2 to ≥4/mm2), and tumor necrosis (score 0 for <10%; 1 for ≥10%). Patients are classified into low risk (0–3 points), intermediate risk (4–5 points), and high risk (6–7 points) (78).

The primary treatment for SFTs is surgical excision; adjuvant radiotherapy or chemotherapy is usually unnecessary. Due to the rarity of SFTs and the need for randomized clinical trials, there is no global consensus on management, and a multidisciplinary treatment approach is recommended. In managing retroperitoneal SFTs specifically, the goal is to achieve negative margins and complete tumor excision, ideally including an adjacent adipose tissue margin, to ensure complete resection and minimize morbidity. Approximately 10% of cases that present more aggressive characteristics, such as a size greater than 10 cm or histological markers of malignancy, may require a more aggressive surgical approach, including the resection of adjacent organs (46, 76).

## Malignant peripheral nerve sheath tumors

Malignant peripheral nerve sheath tumors (MPNST) account for approximately 10% of all soft tissue sarcomas and develop in 8-13% of patients with neurofibromatosis type 1 (NF1), representing one of the primary causes of mortality in these patients. MPNSTs can also arise sporadically or following radiation exposure. These tumors are known for their aggressiveness and invasiveness, with a high rate of local and systemic recurrence, often exhibiting characteristics similar to leiomyosarcomas (LMS). Genetic instability, a hallmark of MPNSTs, includes nucleotide sequence mutations, microsatellite instability, and significant chromosomal alterations such as gains, losses, and rearrangements leading to DNA copy number changes (CNAs). Recurrent losses in numerous chromosomal regions, such as 1p, 9p, 11, 12p, 14q, and 22q, are expected, while typical gains occur on chromosomes 7, 8q, 9q, 13q, 15q, and 17q (79). Diagnosis is supported by imaging techniques such as magnetic resonance imaging (MRI), which is used to locate the tumor, determine its size, and assess its invasiveness. Although MRI and computed tomography (CT) are not entirely reliable in detecting malignant transformation, 18-fluorodeoxyglucose positron emission tomography (FDG-PET) can identify increased metabolism in malignant tumors, facilitating discrimination between MPNST and benign plexiform neurofibromas (pNF). FDG-PET, when combined with CT or MRI, offers superior capability in differentiating benign from malignant lesions and estimating the degree of malignancy in heterogeneous lesions (79, 80).

The primary treatment for MPNST is complete resection with negative margins (R0), which aims to preserve adjacent structures and minimize associated morbidity. However, resection of MPNSTs typically involves removal of the nerve of origin, entailing significant surgical morbidity. Despite these challenges, R0 resection remains the standard of care and the only option with curative potential, given the limited efficacy of available alternative therapies. These tumors, often originating in neural structures such as the femoral nerve in the retroperitoneum, present significant post-resection functional loss. It is crucial for physicians to discuss the potential impact of surgery with patients beforehand (56, 79).

The recommended approach for treating MPNST is multimodal. Although there is no evidence that extended, resections improve outcomes, complete surgical resection offers the best chance of cure. Indeed, 80% of patients who achieve complete removal survive at ten years, compared to 14% of those with incomplete removal. Overall survival varies based on several factors; it is lower in the presence of NF1, decreases with incomplete surgical resection, and is affected by synchronous metastases. Furthermore, larger primary tumors are associated with lower survival. In managing these high-risk tumors, the surgical approach is frequently combined with other treatment modalities, such as radiotherapy and chemotherapy, to improve both local and systemic disease control. This integrated approach is essential, given that local recurrence and distant metastases are the leading causes of morbidity and mortality in patients with high-grade RPS (56, 79, 81).

**Table 1**, which details the surgical management and outcomes associated with each significant RPS histology, underscores the need to adopt a therapeutic strategy based on the tumor’s specific biology, aiming to maximize both survival and postoperative quality of life.

## Morbidity in the management of RPS

The management of RPS involves a significant profile of morbidity and mortality, mainly due to the need for extensive multivisceral surgery. Case series have documented a severe 30-day postoperative morbidity (Clavien-Dindo ≥ 3) of 16.4% and a mortality of 1.8%, with hemorrhages, anastomotic leaks, and abscesses being the most common complications. Identified risk factors include the patient’s advanced age, the need for intraoperative transfusions, and the number of organs resected. Interventions that carry higher risks include caudal pancreaticoduodenectomy, significant vascular resections, splenectomies, and pancreatectomies. Despite the association of surgical morbidity with complex procedures, recent studies have shown a decrease in postsurgical morbidity, even with an increase in the number of organs resected. This improvement could reflect advancements in perioperative care and patient selection. However, surgical morbidity has not demonstrated a direct correlation with local or distant recurrence or overall survival (34).

In terms of long-term morbidity, research has evaluated consequences such as renal failure, chronic pain, and functional deficiencies, finding that serious complications related to femoral nerve resections are infrequent. No significant differences were observed in postoperative creatinine levels between patients with and without nephrectomy, indicating minimal impact on renal and adrenal function from nephrectomy and adrenalectomy. Surgical treatment of RPS may require resection of iliac or femoral vessels to achieve negative margins, often followed by prosthetic vascular reconstructions. These interventions increase postoperative morbidity and mortality but are essential for maintaining vascular integrity. In cases where the involvement of prominent pelvic veins has generated robust collateral venous circulation, resection without reconstruction may be appropriate, thus avoiding the need for venous stents. Lymphadenectomy is justified only in the presence of evidence of lymphatic spread. Interventions involving significant vessels or pancreaticoduodenectomy are linked with the highest risk of serious complications. Patients undergoing these procedures have shown increased ICU admissions (83% vs. 33%; p=0.0257) and higher incidence of significant complications, most frequently pancreatic fistula, associated with a mortality rate of 3.4%. Other long-term complications include the development of incisional hernias (16.8%), alterations in excretion (41%), changes in urinary habits (9%), erectile dysfunction (27.3%), retrograde ejaculation (9%), and dyspareunia (22%). The selection of therapeutic strategies must be individualized, weighing the balance between the benefits of radical resection and the risks of associated morbidity to preserve the patient’s quality of life (33, 35, 46, 71).

## Radiotherapy

The role of radiotherapy in treating primary RPS remains a topic of intense debate despite its recognized benefit in locally controlling extremity sarcomas. The application of RT in RPS is particularly complicated by anatomical and biological differences specific to the retroperitoneum. Preoperative radiotherapy is preferred because it allows better delineation of the target volume, takes advantage of greater tissue oxygenation, and facilitates tumor detachment from vital organs, thus reducing the risk of local recurrence—a significant cause of therapeutic failure (81, 82). The STRASS-1 study, a multicenter randomized phase III trial, showed no improvement in abdominal recurrence-free survival (ARFS) with the addition of preoperative RT compared to surgery alone. However, additional analyses indicate that in specific subgroups, such as patients with low-grade well-differentiated and dedifferentiated liposarcoma, RT may significantly reduce local recurrence. These findings underscore the importance of considering particular histology when evaluating the potential benefits of RT. Technical challenges of RT include precision in tumor volume delineation, which is essential for its effectiveness. Common planning errors, such as deviations in the delineation of the macroscopic tumor volume, could have compromised the results of the STRASS study (83).

Additionally, the trial needed to adequately stratify patients by histology, which may have affected the interpretation of the results. The results were revealed in a follow-up study with quality-adjusted analysis derived from STRASS-1, where treatments were classified as radiotherapy-compliant (RC) or non-radiotherapy-compliant (NRC) for patients with unacceptable deviations. The 3-year ARFS rate was 66.8% (95% CI, 55.8%-75.7%) for the RC group and 49.8% (95% CI, 32.7%-64.8%) for the NRC group, respectively (adjusted hazard ratio, 2.32; 95% CI, 1.25-4.32; P = .008); local recurrence after macroscopic complete resection occurred in 13 of 89 patients (14.6%) in the RC group versus 2 of 36 patients (5.6%) in the NRC group (84).

More recent studies, such as STRASS and STREXIT, which employed propensity-adjusted analyses, found a significant improvement in ARFS for patients with low-grade dedifferentiated liposarcoma treated with preoperative RT, suggesting that RT may have a more prominent role in specific histological subtypes. Although RT remains vital in managing RPS, its application must be carefully considered and tailored according to tumor histology. Future clinical trials should focus on stratifying patients by histologic type and prospectively examining the benefits of RT, especially in subgroups that may derive more significant benefits. This will help refine the role of RT and optimize outcomes for patients with RPS, leading toward more personalized and effective treatment. Furthermore, RT for RPS has shown potential benefits in local control in multiple retrospective series, although these results have been questioned due to possible selection biases. Unfortunately, the difficulty in enrolling patients in randomized trials was confirmed with the premature closure of the ACSOG Z9031 trial 2014. The most robust evidence comes from the phase III STRASS trial, which evaluated radiotherapy in a neoadjuvant setting. The final results were negative, with no difference in ARFS between the surgery arms with and without preoperative RT (28, 81, 85, 86).

Proponents of RT for RPS highlight that RT was associated with a significant reduction of more than 50% in local relapse in all patients. However, approximately 25% of RT plans had significant deviations related to inadequate delineation of the macroscopic tumor volume, which may have affected the results. Furthermore, the lack of robust stratification by histology in the trial could have influenced the interpretation of the data. A *post hoc* exploratory sensitivity analysis found that patients with WDLS and DDLS G1-2 improved abdominal recurrence-free survival (HR 0.62, 95% CI: 0.38–1.02) with statistical significance. Using a propensity-matched analysis, a recent study combined patients treated under the STRASS protocol and those not treated in a study known as STREXIT. This analysis showed significantly improved abdominal recurrence-free survival with preoperative RT in patients with liposarcoma (83, 87).

## Systemic therapy

In the neoadjuvant or adjuvant setting, and even in combination with radiotherapy, chemotherapy has an established role in the treatment of soft tissue sarcomas (STS). In RPS management, chemotherapy remains an area of notable uncertainty, mainly due to anatomical and biological differences that complicate the direct extrapolation of data from soft tissue sarcomas of the extremities and trunk (27, 46). Given this uncertainty, patient participation in available clinical trials is recommended. The ongoing phase III randomized controlled trial, STRASS 2, estimated to be completed by 2029, evaluates a histology-tailored chemotherapy regimen in patients with leiomyosarcoma and dedifferentiated liposarcoma at high risk for distant metastatic recurrence. This study compares neoadjuvant chemotherapy followed by surgery to surgery alone, aiming to improve disease control and survival. Preliminary findings and previous retrospective studies have shown conflicting results, reflecting the complexity of chemotherapy’s impact on these patients (40).

The development of systemic treatments in the field of sarcomas faces the challenge of histological heterogeneity, with varied biologies and responses to treatment. Tseng et al. observed that tumor responses varied depending on the histological type and the chemotherapy regimen. Specifically, patients with leiomyosarcoma (LMS) who received doxorubicin and dacarbazine showed a partial response rate of 37%, compared with only 16% for those who received another chemotherapy combination. This finding aligns with other retrospective studies indicating that ifosfamide has limited activity in LMS compared to dacarbazine (88).

In some reference centers, neoadjuvant chemotherapy is standard for histologies such as high-grade dedifferentiated liposarcoma and leiomyosarcoma, which present high-risk criteria for distant recurrence. For cases of resectable but high-risk RPS (SARCULATOR OS < 60% at ten years), neoadjuvant chemotherapy is considered with the primary goal of increasing overall survival (OS) and, secondarily, of reducing tumor size to facilitate surgery (89). A recent multi-institutional retrospective study revealed that 23% of patients with RPS had a RECIST partial response (>30% tumor reduction) to neoadjuvant chemotherapy. In comparison, 21% showed disease progression associated with significantly worse survival. This suggests that response to treatment could be a criterion for evaluating the appropriateness of proceeding with resection (40).

Finally, the combination of chemotherapy and preoperative radiotherapy (chemoradiation) in patients with RPS remains an experimental approach. Prospective studies are required to determine whether preoperative chemoradiation offers advantages over radiotherapy alone.

The specific application of chemotherapy in RPS needs to be clarified. The treatment strategy for RPS must be carefully evaluated and personalized, prioritizing participation in clinical trials that can provide additional insights and optimize interventions for this complex disease.

## Conclusions

Over the past few years, significant advances have been made in understanding the biology and treatment modalities of RPS. Surgery remains the fundamental pillar and the only curative treatment for localized disease. Meticulous surgical planning is crucial and must be personalized based on specific factors such as tumor histology, location, extension, high-risk characteristics, patient age, comorbidities, and tumor biology. The goal is to standardize the surgical approach to optimize the chances of achieving a complete resection. Global collaboration and specialization of sarcoma teams have increased disease-free and overall survival rates for patients with resected RPS. These advances have facilitated a better understanding of the disease and the development of more personalized treatment strategies, marking a paradigm shift in patients’ prognosis and quality of life. Improvements in the quality of oncologic surgery, appropriate patient selection, and enhancements in perioperative management, including neoadjuvant therapy and intraoperative radiotherapy, are crucial in this progress.

Managing this diverse group of tumors is complex and requires recognizing the multifaceted aspects of surgical management, which must extend beyond mere resection. The goal is always to achieve a complete en-bloc resection, maximizing disease clearance while balancing the associated morbidity and thoroughly understanding the expected post-surgery outcomes based on the tumor’s histologic type. The treatment of RPS is constantly evolving, and new research findings will influence future guidelines and clinical practices, providing a more substantial basis for decision-making. Continued research is essential to further our understanding and management of RPS. Basic and translational research focused on RPS biology and global collaborative efforts are crucial to accelerate progress in this field. The Australasian Transatlantic Retroperitoneal Sarcoma Working Group (TARPSWG) has catalyzed critical retrospective and prospective studies, demonstrating the value of multicenter collaboration in advancing the knowledge and treatment of this rare and challenging disease.

## References

[1] Burningham Z Hashibe M Spector L Schiffman JD. The epidemiology of sarcoma. Clin Sarcoma Res. (2012) 2:14. doi: 10.1186/2045-3329-2-14 23036164 PMC3564705

[2] Fabiano S Contiero P Barigelletti G D’Agostino A Tittarelli A Mangone L. Epidemiology of soft tissue sarcoma and bone sarcoma in Italy: analysis of data from 15 population-based cancer registries. Sarcoma. (2020) 2020(1):6142613. doi: 10.1155/2020/6142613

[3] Reichardt P. Soft tissue sarcomas, a look into the future: Different treatments for different subtypes. Future Oncol. (2014) 10(sup8):s19–27. 25048045 10.2217/fon.14.116

[4] Petrou A Constantinidou A Kontos M Papalampros A Moris D Bakoyiannis C. Comprehensive surgical treatment as the mainstay of management in retroperitoneal sarcomas: Retrospective study from two non-sarcoma specialist centers. Anticancer Res. (2017) 37:2025–31. doi: 10.21873/anticanres.11547 28373477

[5] Porter GA Baxter NN Pisters PWT. Retroperitoneal sarcoma: A population-based analysis of epidemiology, surgery, and radiotherapy. Cancer. (2006) 106:1610–6. doi: 10.1002/cncr.v106:7 16518798

[6] Lewis JJ Leung D Woodruff JM Brennan MF. Retroperitoneal soft-tissue sarcoma: Analysis of 500 patients treated and followed at a single institution. Ann Surg. (1998) 228(3):355–65. 10.1097/00000658-199809000-00008 PMC1191491 9742918

[7] Gronchi A Miceli R Allard MA Callegaro D Le Péchoux C Fiore M. Personalizing the approach to retroperitoneal soft tissue sarcoma: histology-specific patterns of failure and postrelapse outcome after primary extended resection. Ann Surg Oncol. (2015) 22:1447–54. doi: 10.1245/s10434-014-4130-7 25300609

[8] Munoz P Bretcha-Boix P Artigas V Asencio JM. Surgical principles of primary retroperitoneal sarcoma in the era of personalized treatment: A review of the frontline extended surgery. Cancers. (2022) 14:4091. doi: 10.3390/cancers14174091 36077627 PMC9454716

[9] Gronchi A Miceli R Colombo C Stacchiotti S Collini P Mariani L. Frontline extended surgery is associated with improved survival in retroperitoneal low- to intermediate-grade soft tissue sarcomas. Ann Oncol. (2012) 23:1067–73. doi: 10.1093/annonc/mdr323 21765179

[10] Bonvalot S Miceli R Berselli M Causeret S Colombo C Mariani L. Aggressive surgery in retroperitoneal soft tissue sarcoma carried out at high-volume centers is safe and is associated with improved local control. Ann Surg Oncol. (2010) 17:1507–14. doi: 10.1245/s10434-010-1057-5 20393803

[11] Smith HG Panchalingam D Hannay JAF Smith MJF Thomas JM Hayes AJ. Outcome following resection of retroperitoneal sarcoma. Br J Surg. (2015) 102:1698–709. doi: 10.1002/bjs.9934 26395577

[12] Garcia-Ortega DY Jiménez-Ortega JA Melendez-Fernandez AP Álvarez-Cano A Caro-Sanchez CHS Vargas-Lara AK. Does compartmental resection really impact retroperitoneal soft tissue sarcomas? A retrospective analysis from a Single Referral Center. Surg Oncol. (2023) 51:101997. 37832278 10.1016/j.suronc.2023.101997

[13] Tseng WW Seo HJ Pollock RE Gronchi A. Historical perspectives and future directions in the surgical management of retroperitoneal sarcoma. Surg Oncol. (2018) 117:7–11. doi: 10.1002/jso.24888 29127700

[14] Armstrong JR Cohn I. Primary Malignant retroperitoneal tumors. Am J Surg. (1965) 110:937–43. doi: 10.1016/0002-9610(65)90181-9 5891845

[15] Quagliuolo V Ruspi L Cananzi FCM Gronchi A. History of surgery in retroperitoneal sarcomas. Updates Surg. (2019), 1–7. doi: 10.1007/978-88-470-3980-3_1

[16] Paik B Seo CJ Tan JWS Juan WKD Soo KC Ong CAJ. A systematic review of margin status in retroperitoneal liposarcomas: Does the R0 margin matter? Front Oncol. (2022) 12. doi: 10.3389/fonc.2022.891710 PMC9404241 36033535

[17] Storm FK Mahvi DM. Diagnosis and management of retroperitoneal soft-tissue sarcoma. Ann Surg. (1991) 214:2–10. doi: 10.1097/00000658-199107000-00002 2064467 PMC1358407

[18] Trans-Atlantic RPS Working Group. Management of primary retroperitoneal sarcoma (RPS) in the adult: A consensus approach from the trans-atlantic RPS working group. Ann Surg Oncol. (2015) 22:256–63. doi: 10.1245/s10434-014-3965-2 25316486

[19] Musa J Willis F Harnoss JM Rompen IF Sauerteig C Kochendoerfer SM. A century of retroperitoneal soft-tissue sarcoma research: From single center experience to precision oncology? A bibliometric analysis of past, present, and future perspectives. Eur J Surg Oncol. (2023) 49(9):106948. 37286428 10.1016/j.ejso.2023.05.023

[20] Álvarez Álvarez R Manzano A Agra Pujol C Artigas Raventós V Correa R Cruz Jurado J. Updated review and clinical recommendations for the diagnosis and treatment of patients with retroperitoneal sarcoma by the spanish sarcoma research group (GEIS). Cancers (Basel). (2023) 15(12):3194. doi: 10.3390/cancers15123194 37370803 PMC10295927

[21] Tseng WW Swallow CJ Strauss DC Bonvalot S Rutkowski P Ford SJ. Management of locally recurrent retroperitoneal sarcoma in the adult: an updated consensus approach from the transatlantic australasian retroperitoneal sarcoma working group. Ann Surg Oncol. (2022) 29:7335–48. doi: 10.1245/s10434-022-11864-y 35767103

[22] Swallow CJ Strauss DC Bonvalot S Rutkowski P Desai A Gladdy RA. Management of primary retroperitoneal sarcoma (RPS) in the adult: an updated consensus approach from the transatlantic australasian RPS working group. Ann Surg Oncol. (2021) 28:7873–88. doi: 10.1245/s10434-021-09654-z PMC9257997 33852100

[23] Webster S Vargas AC Maclean F Vu J Tong E Coker D. What is the association of preoperative biopsy with recurrence and survival in retroperitoneal sarcoma? A systematic review by the Australia and New Zealand Sarcoma Association clinical practice guidelines working party. Crit Rev Oncol Hematol. (2024) 197:104354. doi: 10.1016/j.critrevonc.2024.104354 38614268

[24] Sassa N Yokoyama Y Nishida Y Yamada S Uchida H Kajiyama H. Clinical characteristics and surgical outcomes of retroperitoneal tumors: a comprehensive data collection from multiple departments. Int J Clin Oncol. (2020) 25:929–36. doi: 10.1007/s10147-020-01620-1 31950376

[25] Wilkinson MJ Martin JL Khan AA Hayes AJ Thomas JM Strauss DC. Percutaneous core needle biopsy in retroperitoneal sarcomas does not influence local recurrence or overall survival. Ann Surg Oncol. (2015) 22:853–8. doi: 10.1245/s10434-014-4059-x 25190132

[26] Van Houdt WJ Schrijver AM Cohen-Hallaleh RB Memos N Fotiadis N Smith MJ. Needle tract seeding following core biopsies in retroperitoneal sarcoma. Eur J Surg Oncol. (2017) 43:1740–5. doi: 10.1016/j.ejso.2017.06.009 28754227

[27] Blay JY Hindi N Bollard J Aguiar S Angel M Araya B. SELNET clinical practice guidelines for soft tissue sarcoma and GIST. Cancer Treat Rev. (2022) 102:102312. doi: 10.1016/j.ctrv.2021.102312 34798363

[28] Casali PG Abecassis N Bauer S Biagini R Bielack S Bonvalot S. Soft tissue and visceral sarcomas: ESMO-EURACAN Clinical Practice Guidelines for diagnosis, treatment and follow-up. Ann Oncol. (2018) 29:iv51–67. doi: 10.1093/annonc/mdy096 29846498

[29] Fanciullo C Gitto S Carlicchi E Albano D Messina C Sconfienza LM. Radiomics of musculoskeletal sarcomas: A narrative review. J Imaging. (2022) 8(2):45. doi: 10.3390/jimaging8020045 35200747 PMC8876222

[30] Ghadimi M Bruns CJ. Systematic surgery of retroperitoneal sarcomas: Imaging-guided planning of surgical strategy. Chirurg. (2019) 90:447–56. doi: 10.1007/s00104-019-0952-y 31001643

[31] Rust DJ Kato T Yoon SS. Treatment for local control of retroperitoneal and pelvis sarcomas: A review of the literature. Surg Oncol. (2022) 43:101814. 35834940 10.1016/j.suronc.2022.101814

[32] Kirane A Crago AM. The importance of surgical margins in retroperitoneal sarcoma. J Surg Oncol. (2016) 113:270–6. doi: 10.1002/jso.v113.3 26707028

[33] Ruff SM Grignol VP Contreras CM Pollock RE Beane JD. Morbidity and Mortality after Surgery for Retroperitoneal Sarcoma. Curr Oncol. (2023) 30:492–505. doi: 10.3390/curroncol30010039 PMC9858026 36661688

[34] Macneill AJ Gronchi A Miceli R Bonvalot S Swallow CJ Hohenberger P. Postoperative Morbidity after Radical Resection of Primary Retroperitoneal Sarcoma. Ann Surg. (2018) 267:959–64. doi: 10.1097/SLA.0000000000002250 28394870

[35] Callegaro D Miceli R Brunelli C Colombo C Sanfilippo R Radaelli S. Long-term morbidity after multivisceral resection for retroperitoneal sarcoma. Br J Surg. (2015) 102:1079–87. doi: 10.1002/bjs.9829 26041724

[36] Callegaro D Fiore M Gronchi A. Personalizing surgical margins in retroperitoneal sarcomas. Expert Rev Anticancer Ther. (2015) 15:553–67. doi: 10.1586/14737140.2015.1028375 25797538

[37] Schmitz E Nessim C. Retroperitoneal sarcoma care in 2021. Cancers (Basel). (2022) 14(5):1293. 35267600 10.3390/cancers14051293 PMC8909774

[38] Sassa N. Retroperitoneal tumors: Review of diagnosis and management. Int J Urol. (2020) 27:1058–70. doi: 10.1111/iju.v27.12 32914475

[39] Bonvalot S Raut CP Pollock RE Rutkowski P Strauss DC Hayes AJ. Technical considerations in surgery for retroperitoneal sarcomas: Position paper from E-Surge, a master class in sarcoma surgery, and EORTC-STBSG. Ann Surg Oncol. (2012) 19:2981–91. doi: 10.1245/s10434-012-2342-2 22476756

[40] Delisle M Gyorki D Bonvalot S Nessim C. Landmark series: A review of landmark studies in the treatment of primary localized retroperitoneal sarcoma. Ann Surg Oncol. (2022) 29:7297–311. doi: 10.1245/s10434-022-12517-w 36088426

[41] Faron M Cavalcanti A Honore C. Compartmental resection of a retroperitoneal sarcoma. J Visc Surg. (2019) 156:245–51. doi: 10.1016/j.jviscsurg.2019.02.002 31130355

[42] Guo Q Zhao J Du X Huang B. Survival outcomes of surgery for retroperitoneal sarcomas: A systematic review and meta-analysis. PloS One. (2022) 17(7):e0272044. doi: 10.1371/journal.pone.0272044 35901187 PMC9333279

[43] Gronchi A Lo Vullo S Fiore M Mussi C Stacchiotti S Collini P. Aggressive surgical policies in a retrospectively reviewed single-institution case series of retroperitoneal soft tissue sarcoma patients. J Clin Oncol. (2009) 27:24–30. doi: 10.1200/JCO.2008.17.8871 19047283

[44] Bonvalot S Rivoire M Castaing M Stoeckle E Le Cesne A Blay JY. Primary retroperitoneal sarcomas: A multivariate analysis of surgical factors associated with local control. J Clin Oncol. (2009) 27:31–7. doi: 10.1200/JCO.2008.18.0802 19047280

[45] Stahl CC Schwartz PB Ethun CG Marka N Krasnick BA Tran TB. Renal function after retroperitoneal sarcoma resection with nephrectomy: A matched analysis of the United States sarcoma collaborative database. Ann Surg Oncol. (2021) 28:1690–6. doi: 10.1245/s10434-020-09290-z PMC7897241 33146839

[46] Nessim C Raut CP Callegaro D Barretta F Miceli R Fairweather M. Postoperative morbidity after resection of recurrent retroperitoneal sarcoma: A report from the transatlantic australasian RPS working group (TARPSWG). Ann Surg Oncol. (2021) 28:2705–14. doi: 10.1245/s10434-020-09445-y 33389288

[47] Improta L Pasquali S Iadecola S Barisella M Fiore M Radaelli S. Organ infiltration and patient risk after multivisceral surgery for primary retroperitoneal liposarcomas. Ann Surg Oncol. (2023) 30:4500–10. doi: 10.1245/s10434-023-13314-9 36930371

[48] Willis F Buck L Musa J Hinz U Mechtersheimer G Seidensaal K. Long-term quality of life after resection of retroperitoneal soft tissue sarcoma. Eur J Surg Oncol. (2023) 49:106977. doi: 10.1016/j.ejso.2023.07.003 37481390

[49] Zhuang A Fang Y Ma L Yang H Lu W Zhou Y. Does aggressive surgery mean worse quality of life and functional capacity in retroperitoneal sarcoma patients?—A retrospective study of 161 patients from China. Cancers (Basel). (2022) 14(20):5126. doi: 10.3390/cancers14205126 36291911 PMC9600768

[50] Tseng WW Tsao-Wei DD Callegaro D Grignani G D’Ambrosio L Bonvalot S. Pancreaticoduodenectomy in the surgical management of primary retroperitoneal sarcoma. Eur J Surg Oncol. (2018) 44:810–5. doi: 10.1016/j.ejso.2018.01.086 29452860

[51] Spolverato G Chiminazzo V Lorenzoni G Fiore M Radaelli S Sanfilippo R. Oncological outcomes after major vascular resections for primary retroperitoneal liposarcoma. Eur J Surg Oncol. (2021) 47:3004–10. doi: 10.1016/j.ejso.2021.06.035 34364722

[52] Kim KD Lee KW Lee JE Hwang JA Jo SJ Kim J. Postoperative outcomes of distal pancreatectomy for retroperitoneal sarcoma abutting the pancreas in the left upper quadrant. Front Oncol. (2021) 11. doi: 10.3389/fonc.2021.792943 PMC8721218 34988024

[53] Asencio Pascual JM Fernandez Hernandez JA Blanco Fernandez G Muñoz Casares C Álvarez Álvarez R Fox Anzorena B. Update in pelvic and retroperitoneal sarcoma management: the role of compartment surgery. Cirugía Española (English Edition). (2019) 97:480–8. doi: 10.1016/j.ciresp.2019.06.011 31521244

[54] Tan MCB Yoon SS. Surgical management of retroperitoneal and pelvic sarcomas. J Surg Oncol. (2015) 111:553–61. doi: 10.1002/jso.23840 PMC4376657 25482329

[55] Gronchi A Strauss DC Miceli R Bonvalot S Swallow CJ Hohenberger P. Variability in patterns of recurrence after resection of primary retroperitoneal sarcoma (RPS): A report on 1007 patients from the multi-institutional collaborative RPS working group. Ann Surg. (2016) 263:1002–9. doi: 10.1097/SLA.0000000000001447 26727100

[56] Dingley B Fiore M Gronchi A. Personalizing surgical margins in retroperitoneal sarcomas: an update. Expert Rev Anticancer Ther. (2019) 19:613–31. doi: 10.1080/14737140.2019.1625774 31159625

[57] Callegaro D Raut CP Ng D Strauss DC Honoré C Stoeckle E. Has the Outcome for Patients Who Undergo Resection of Primary Retroperitoneal Sarcoma Changed Over Time? A Study of Time Trends During the Past 15 years. Ann Surg Oncol. (2021) 28:1700–9. doi: 10.1245/s10434-020-09065-6 33073340

[58] Gronchi A Miceli R Shurell E Eilber FC Eilber FR Anaya DA. Outcome prediction in primary resected retroperitoneal soft tissue sarcoma: histology-specific overall survival and disease-free survival nomograms built on major sarcoma center data sets. J Clin Oncol. (2013) 31:1649–55. doi: 10.1200/JCO.2012.44.3747 23530096

[59] de Bree E Michelakis D Heretis I Kontopodis N Spanakis K Lagoudaki E. Retroperitoneal soft tissue sarcoma: emerging therapeutic strategies. Cancers (Basel). (2023) 15(22):5469. 38001729 10.3390/cancers15225469 PMC10670057

[60] Blay JY Soibinet P Penel N Bompas E Duffaud F Stoeckle E. Improved survival using specialized multidisciplinary board in sarcoma patients. Ann Oncol. (2017) 28:2852–9. doi: 10.1093/annonc/mdx484 PMC5834019 29117335

[61] Bonvalot S Gaignard E Stoeckle E Meeus P Decanter G Carrere S. Survival benefit of the surgical management of retroperitoneal sarcoma in a reference center: A nationwide study of the french sarcoma group from the netSarc database. Ann Surg Oncol. (2019) 26:2286–93. doi: 10.1245/s10434-019-07421-9 31065964

[62] Tyler R Wanigasooriya K Taniere P Almond M Ford S Desai A. A review of retroperitoneal liposarcoma genomics. Cancer Treat Rev. (2020) 86:102013. 32278233 10.1016/j.ctrv.2020.102013

[63] Thway K. Well-differentiated liposarcoma and dedifferentiated liposarcoma: An updated review. Semin Diagn Pathol. (2019) 36:112–21. doi: 10.1053/j.semdp.2019.02.006 30852045

[64] Dei Tos AP. Liposarcoma: New entities and evolving concepts. Ann Diagn Pathol. (2000) 4:252–66. doi: 10.1053/adpa.2000.8133 10982304

[65] Setsu N Miyake M Wakai S Nakatani F Kobayashi E Chuman H. Primary retroperitoneal myxoid liposarcomas. Am J Surg Pathol. (2016) 40:1286. doi: 10.1097/PAS.0000000000000657 27158758 PMC5029446

[66] Dumitru DA Bejenariu AV Marin A Costache A Pleomorphic M Ciongariu AM. Pleomorphic liposarcoma unraveled: investigating histopathological and immunohistochemical markers for tailored diagnosis and therapeutic innovations. Medicina. (2024) 60:950. doi: 10.3390/medicina60060950 38929567 PMC11205576

[67] Mastoraki A Leotsakos G Mastoraki S Papanikolaou IS Danias N Smyrniotis V. Challenging diagnostic and therapeutic modalities for leiomyosarcoma of inferior vena cava. Int J Surg. (2015) 13:92–5. doi: 10.1016/j.ijsu.2014.11.051 25489949

[68] Wang MX Menias CO Elsherif SB Segaran N Ganeshan D. Current update on IVC leiomyosarcoma. Abdominal Radiol. (2021) 46:5284–96. doi: 10.1007/s00261-021-03256-9 34415408

[69] Alkhalili E Greenbaum A Langsfeld M Marek J Rana MA Glew R. Leiomyosarcoma of the inferior vena cava: A case series and review of the literature. Ann Vasc Surg. (2016) 33:245–51. doi: 10.1016/j.avsg.2015.10.016 26802297

[70] Teixeira FJR Netto SD do C Perina AL de F Torricelli FCM Teixeira LR Zerati AE. Leiomyosarcoma of the inferior vena cava: Survival rate following radical resection. Oncol Lett. (2017) 14:3909–16. doi: 10.3892/ol.2017.6706 PMC5651407 29098019

[71] Palacios AR Schmeusser BN Midenberg E Patil D Xie L Nabavizadeh R. Resection of retroperitoneal tumors with inferior vena cava involvement without caval reconstruction. J Surg Oncol. (2022) 126:1306–15. doi: 10.1002/jso.27052 35943295

[72] Wiseman JT Grignol V. Location, location, location: approaches to retroperitoneal vascular leiomyosarcoma. Oncol (Williston Park). (2020) 34:7–10. 32645198

[73] Nooromid M De Martino R Squizzato F Benedetto F De Caridi G Chou EL. Surgical resection and graft replacement for primary inferior vena cava leiomyosarcoma: A multicenter experience. J Vasc Surg Venous Lymphat Disord. (2022) 10:617–25. doi: 10.1016/j.jvsv.2021.06.021 34271247

[74] Tseng WW Barretta F Fiore M Colombo C Radaelli S Baia M. Extent of macroscopic vascular invasion predicts distant metastasis in primary leiomyosarcoma of the inferior vena cava. J Surg Oncol. (2024). doi: 10.1002/jso.27799 39155701

[75] Klemperer P Coleman BR. Primary neoplasms of the pleura. A report of five cases. Am J Ind Med. (1992) 22:4–31. doi: 10.1002/ajim.4700220103 1415270

[76] Tariq MU Din NU Abdul-Ghafar J Park YK. The many faces of solitary fibrous tumor; diversity of histological features, differential diagnosis and role of molecular studies and surrogate markers in avoiding misdiagnosis and predicting the behavior. Diagn Pathol. (2021) 16. doi: 10.1186/s13000-021-01095-2 PMC8059036 33879215

[77] Choi JH Ro JY. The 2020 WHO classification of tumors of soft tissue: selected changes and new entities. Adv Anat Pathol. (2021) 28(1):44–58. 32960834 10.1097/PAP.0000000000000284

[78] Demicco EG Wagner MJ Maki RG Gupta V Iofin I Lazar AJ. Risk assessment in solitary fibrous tumors: validation and refinement of a risk stratification model. Mod Pathol. (2017) 30:1433–42. doi: 10.1038/modpathol.2017.54 28731041

[79] Somatilaka BN Sadek A McKay RM Le LQ. Malignant peripheral nerve sheath tumor: models, biology, and translation. Oncogene. (2022) 41:2405. doi: 10.1038/s41388-022-02290-1 35393544 PMC9035132

[80] Tovmassian D Abdul Razak M London K. The role of [18F]FDG-PET/CT in predicting Malignant transformation of plexiform neurofibromas in neurofibromatosis-1. Int J Surg Oncol. (2016) 2016(1):6162182. 28058117 10.1155/2016/6162182 PMC5183794

[81] Othman H Shapiro J Chung P Gladdy RA. Progress in retroperitoneal sarcoma management: surgical and radiotherapy approaches. Semin Radiat Oncol. (2024) 34:164–71. doi: 10.1016/j.semradonc.2024.02.002 38508781

[82] Zhang M Crago A Yoon SS Singer S Alektiar K. Feasibility of preoperative dose-painting intensity modulated radiation therapy (IMRT) for borderline-resectable primary retroperitoneal sarcoma. Ann Surg Oncol. (2022) 29:7115–21. doi: 10.1245/s10434-022-12053-7 PMC10367943 35771370

[83] Bonvalot S Gronchi A Le Pechoux C Swallow CJ Strauss DC Meeus P. STRASS (EORTC 62092): A phase III randomized study of preoperative radiotherapy plus surgery versus surgery alone for patients with retroperitoneal sarcoma. J Clin Oncol. (2019) 37:11001–1. doi: 10.1200/JCO.2019.37.15_suppl.11001

[84] Haas R Stelmes JJ Zaffaroni F Sauvé N Clementel E Bar-Deroma R. Critical impact of radiotherapy protocol compliance and quality in the treatment of retroperitoneal sarcomas: Results from the EORTC 62092-22092 STRASS trial. Cancer. (2022) 128:2796–805. doi: 10.1002/cncr.34239 35536104

[85] Nussbaum DP Rushing CN Lane WO Cardona DM Kirsch DG Peterson BL. Preoperative or postoperative radiotherapy versus surgery alone for retroperitoneal sarcoma: a case-control, propensity score-matched analysis of a nationwide clinical oncology database. Lancet Oncol. (2016) 17:966–75. doi: 10.1016/S1470-2045(16)30050-X 27210906

[86] Smith MJF Ridgway PF Catton CN Cannell AJ O’Sullivan B Mikula LA. Combined management of retroperitoneal sarcoma with dose intensification radiotherapy and resection: long-term results of a prospective trial. Radiother Oncol. (2014) 110:165–71. doi: 10.1016/j.radonc.2013.10.041 24411227

[87] Callegaro D Raut CP Ajayi T Strauss D Bonvalot S Ng D. Preoperative radiotherapy in patients with primary retroperitoneal sarcoma: EORTC-62092 trial (STRASS) versus off-trial (STREXIT) results. Ann Surg. (2023) 278:127–34. doi: 10.1097/SLA.0000000000005492 35833413

[88] Tseng WW Barretta F Conti L Grignani G Tolomeo F Albertsmeier M. Defining the role of neoadjuvant systemic therapy in high-risk retroperitoneal sarcoma: A multi-institutional study from the Transatlantic Australasian Retroperitoneal Sarcoma Working Group. Cancer. (2021) 127:729–38. doi: 10.1002/cncr.33323 33206381

[89] Pasquali S Colombo C Bottelli S Verderio P Broto JM Lopez–Pousa A. The sarculator predicted risk of distant metastasis and overall survival in patients with high-risk soft tissue sarcoma treated with perioperative chemotherapy in a randomized controlled trial. Eur J Surg Oncol. (2018) 44:e2. doi: 10.1016/j.ejso.2018.07.012

